# Sources of Polonium ^210^Po and Radio-Lead ^210^Pb in Human Body in Poland

**DOI:** 10.3390/ijerph19041984

**Published:** 2022-02-10

**Authors:** Alicja Boryło, Bogdan Skwarzec, Jarosław Wieczorek

**Affiliations:** Department of Chemistry and Environmental Radiochemistry, Faculty of Chemistry, University of Gdańsk, 80-308 Gdańsk, Poland; bogdan.skwarzec@ug.edu.pl (B.S.); jaroslaw.wieczorek@ug.edu.pl (J.W.)

**Keywords:** radionuclides, polonium ^210^Po and radio-lead ^210^Pb, human body

## Abstract

This article contains and discusses the results of research on the source of polonium ^210^Po and radio-lead ^210^Pb in the human body of adults living in Poland. An adult inhabitant of Poland receives an effective annual radiation dose of 309 µSv from inhalation and absorption of ^210^Po and ^210^Pb. The main sources of both radionuclides in the body is cigarette and marijuana smoking. In terms of food, the consumption of fish, cereals, vegetables and fruit as well as mushrooms have the largest contribution to annual dose. This study highlights the importance of cigarette smoking and the growing importance of marijuana hash smoking as the main source of ^210^Po and ^210^Pb for adults living in Poland. The calculated dose that results from the decay of both radionuclides in body is 1/10 of the annual radiation dose received by a Polish inhabitant from natural sources (2.8 mSv) and is almost five times lower than the dose resulting from the inhalation of ^222^Rn.

## 1. Introduction

The long-term effects of the radionuclide intake by the human body are the most important from the radiochemical and radiological point of view. A large contribution towards the radiation dose received by humans comes from the naturally occurring uranium found in series of radionuclides accumulated in the body, namely alpha emitting ^210^Po (of physical half-life 138.4 days) and ^210^Pb (precursor of ^210^Po with physical half-life 22.2 years) [1]. The specific ability of biota (especially marine) to accumulate polonium leads to their exposure to an alpha radiation dose [2,3,4,5,6]. The report of the United Nations Scientific Committee on the Effects of Atomic Radiation indicated that ^210^Po is estimated to contribute about 7% of the total effective dose to man from ingested natural internal radiation [1]. The polonium isotopes are amongst the most radiotoxic nuclides to human beings [7]. According to the ICRP model [8] for adults, 10% of the inhaled and 50% of the ingested ^210^Po enter the circulatory system, while the remaining fraction stays at the gastrointestinal system for 24–36 h before being removed by the organisms. The absorbed ^210^Po tends to be accumulated in the liver (30%), kidney (10%), spleen (7%) and bone marrow (10%). The maximum permissible human body-burden for ingested ^210^Po is only 1.1·10^3^ Bq, and the maximum allowable concentration for soluble polonium compounds in the air is about 0.74 Bq/m^3^ [9]. The main sources of ^210^Po (and also ^210^Pb) in the human body are food (fish, meat, cereals and vegetables) as well as tobacco and marijuana hash in the case of smokers [7,10,11]. 

The general source of ^210^Po and ^210^Pb in the atmosphere is ^222^Rn decay within continental landmasses [12,13]. The radionuclides fall out in rain and snow and are then deposited on the land surface (and land plants) and oceans. Atmospheric deposition of ^210^Po and ^210^Pb lies within a broad range from 0.05 to 0.5 kBq/m^2.^y [14]. Polonium uptake by plants occurs indirectly through the root system and directly from the deposition of the fallout onto the plants [15]. In the case of plants with large leaves (tobacco, marijuana, cabbage), direct deposition of ^210^Po and ^210^Pb on the leaf surfaces is the most important route for their accumulation by the plant [16,17,18]. Moreover, the tuberous vegetables (onion, carrot, turnip) are contaminated by ^210^Po and ^210^Pb present in certain fertilizers (particularly phosphate fertilizers) [19,20].

About 400 chemical compounds have been identified in tobacco and marijuana smoke, and it is probable that 70 of them are carcinogenic. The average smoker knows that the compounds contained in the dangerous substances in cigarettes, including dioxins, polycyclic aromatic hydrocarbons (PAH), volatile hydrocarbons and nicotine are probably the reason for the high incidence of cancer in smokers [21,22,23,24,25]. However, less is known of the harmfulness of radioactive isotopes ^210^Po and ^210^Pb contained in marijuana hash [11]. The temperature of the incandescent tobacco and marijuana hash during smoking (400–700 °C) is much higher than the sublimation temperature of lead and polonium. According to sources, 10–15% of radionuclides such as polonium from tobacco are transferred to the lungs during smoking [26]. It is well known that cigarettes and hash marijuana are among the most dangerous environmental factors contributing to the development of cancer, especially of the respiratory system [27,28,29]. According to the World Health Organization, preventing tobacco smoking would be more effective in extending the life and health status of the industrialized population than any other preventive measure [30].

## 2. Materials and Methods

The ^210^Po and ^210^Pb concentrations in the air, water consumption, alcohol (wine, beer) cigarettes, marijuana, honey, herbs and tea, mushrooms, meat (poultry, pork and beef) and fish were determined. After co-precipitation and mineralization of analyzed samples, polonium was separated by electrodeposition onto a silver disc. The measurement of radio-lead ^210^Pb activity of in the analyzed samples can be taken directly by the re-measuring of ^210^Po activity of (after 6–10 months from first deposition), which resulted in the decay of ^210^Pb in the samples [31,32,33]. The annual inhalation of effective radiation doses for cigarettes and marijuana hash smoking was calculated using the conversion factor of 3.3·10^−6^ Sv/Bq for ^210^Po and 1.1·10^−6^ Sv/Bq for ^210^Pb. In the case of water, alcohol (beer, wine), tea, herbs and food, the effective doses were calculated on the basis of the respective conversion factor of 1.2·10^−6^ Sv/Bq for ^210^Po and 0.69·10^−6^ Sv/Bq for ^210^Pb [34].

## 3. Results and Discussion

Intake of ^210^Po and ^210^Pb.

### 3.1. Air Inhalation

The range of activity concentration on ground level air is 0.02–0.3 mBq/m^3^ for ^210^Po and 0.2–1.5 mBq/m^3^ for ^210^Pb [35]. In the air over Poland, both radionuclides are found in quantities towards the lower values of this range [12]. On the basis of the average concentration of ^210^Po and ^210^Pb in the air, and the daily inhalation of air on the volume 24 m^3^, it was concluded that the annual inhalation of both radionuclides from the air by the inhabitants of Poland amounted to 1.63 Bq for ^210^Po and 3.08 Bq for ^210^Po [8]. The annual radiation effective dose calculated on this basis was 5.4 µSv for polonium and 3.6 µSv for radio-lead (Table 1). The air inhalation is a less important source of both radionuclides in the human body and the contribution of air inhaled by Polish citizens accounts for only 0.3% of the total annual natural radiation dose in Poland, which is 2.8 mSv [18]. 

### 3.2. Drinking Water

While consuming drinking water, breathing air and eating food we all ingest natural and artificial radionuclides. Depending on the extent of the contamination in the local environmental and our eating habits, this process occurs with varying degree of intensity. The activity concentration in drinking water is of the order of 0.5–48 mBq/L for ^210^Po and around 1–40 mBq/L for ^210^Pb [35,36]. In Poland, the mean values of ^210^Po and ^210^Pb concentration in drinking water (groundwater, mineral and bottled water) are about 0.51 mBq/L (ranging from 0.2 to 3.4 mBq/L) and 1.60 mBq/dm^3^ (ranging from 0.5 to 10 mBq/L) [37]. The World Health Organization adopted the acceptable alpha emitters (^238^U, ^226^Ra and ^210^Po) below 0.5 Bq/L and the recommended level radiation dose below 0.1 mSv/y for drinking water quality [38]. In Poland, radiochemical analyses of drinking water showed the drinking water is safe from the point of view of radiation protection. On the basis of the average concentration of ^210^Po and ^210^Pb in drinking water, the annual intake of both radionuclides by an adult Pole were calculated (Table 1). For this purpose, the mean ingestion of public water was assumed to be 500 dm^3^ per year. The consumption of drinking water by an average individual in Poland was estimated to be 0.26 Bq/y and 0.81 Bq/y for ^210^Po and ^210^Pb, respectively. The annual effective radiation dose calculated on this basis was 0.8 µSv for both radionuclides. Water ingestion is a less important source of both analyzed radionuclides in the human body, and the contribution of water consumption by Polish citizens accounts for only 0.03% of the total annual radiation dose due to the decay of these radionuclides and only 0.3% of the total absorption of ^210^Po and ^210^Pb by adults living in Poland (Table 1). 

### 3.3. Cigarette and Marijuana Hash Smoking

The content of ^210^Po and ^210^Pb in one twenty-cigarette packet smoked by adults living in Poland ranges from 20 to 215 mBq and depends on the origin, processing and type of tobacco used [18]. Assuming that both the radionuclides in the cigarette are in radioactive equilibrium, it was calculated that by smoking one packet of domestic cigarettes a day, the Polish smokers receive an effective radiation dose exceeding 158 µSv. On the other hand, the number of cigarettes smoked in Poland decreased gradually, from around 100 billion in 1990s to 36 billion in 2020. Smokers make up 25% of the total population (about 8 million people) [33]. This means that a smoker in Poland consumes an average 4.500 cigarettes a year (12 cigarettes a day). If we calculate this for the average inhabitant of Poland, we get 950 cigarettes a year (3 cigarettes a day). If we take into account the real consumption of cigarettes by a smoker in Poland (12 cigarettes a day), the calculated radiation doses are: 70 µSv/y from polonium and 25 µSv/y from radio-lead (total 95 Sv/y), while for an average inhabitant of Poland (3 cigarettes a day) the doses are: 17 µSv/y from ^210^Po and 6 µSv/y from ^210^Pb (total 23 Sv/y). The daily inhalation by adults living in Poland and also other nations is on average more or less 20 times higher than the daily inhalation of atmospheric ^210^Po and ^210^Pb by non-smokers (Table 1). This indicated that cigarette smoking is the main source of ^210^Po and ^210^Pb in the adult population in Poland. On the other hand, radon is an important source of a high dose from primers such as polonium and lead. In Poland, it is estimated that the level of radon in buildings, due to their structures and materials used, is one of the highest in the world, which may have a significant impact on the dose volume in this case [39].

**Figure 1:**
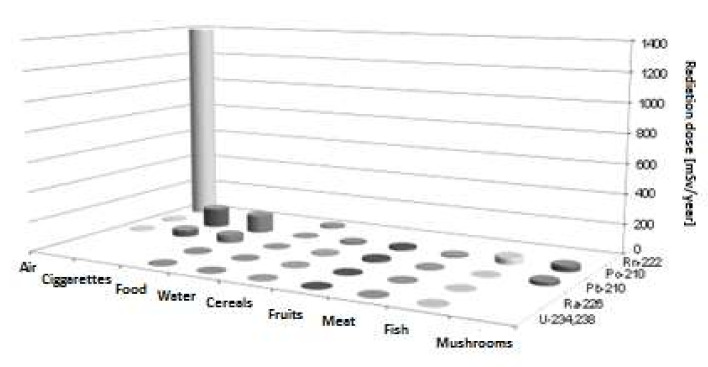
Annual effective radiation dose as a result of the intake of radionuclides by adult inhabitants of Poland (µSv/year).

In the case of marijuana products available on the Polish market, the mean values of ^210^Po and ^210^Pb activity concentration are the following: 12.1 and 3.1 mBq/g in cannabis plant, 4.2 and 0.85 mBq/g in cannabis hemp, 62.4 and 10.5 mBq/g in cannabis hash and 10.9 and 2.3 mBq/g in cannabis tea [11]. The rate of CBD cannabis consumption in Poland is not exactly known; the amount of the annual effective radiation dose used in the calculations was based on the average consumption of CBD cannabis in the USA, which was determined to 1 kg/y [40]. The annual marijuana hash uptake into the lungs of Polish smokers calculated on this basis is 1.02 Bq for ^210^Po and 0.2 Bq for ^210^Pb, and the value of the annual radiation dose from the decay of both radionuclides is 3.6 µSv, but for regular and heavy consumers the annual doses were estimated at 25 and 60 µSv, respectively. The radiation dose originating from inhalation of hash smoke is still much smaller than the effective dose from the intake of radionuclides ^210^Po and ^210^Pb from cigarette smoking, but the increasing consumption of marijuana products in the world is causing the radiation dose to be much greater. Apart from the marijuana hash, the adult inhabitants of Poland also consume tea cannabis, which construes 14.7 µSv to annual radiation dose (Table 1). Our research shows that not only cigarettes, but also hashish smoking and tea cannabis consumption are a source of increased ^210^Po and ^210^Pb radioactivity in the lungs of smokers. The annual radiation doses from smoking in Poland are definitely lower than those from inhalation of ^222^Rn, but higher than the doses resulting from food consumption, mainly fish and mushrooms (Table 1, Figure 1). However, inhalation of ^222^Rn seems to be the main source of radioactive dose in Poland, especially that Polish buildings have one of the highest radon levels for buildings in the world. The main purpose of this publication was to estimate the dose from the consumption of products and smoking.

### 3.4. Food

**Figure 2:**
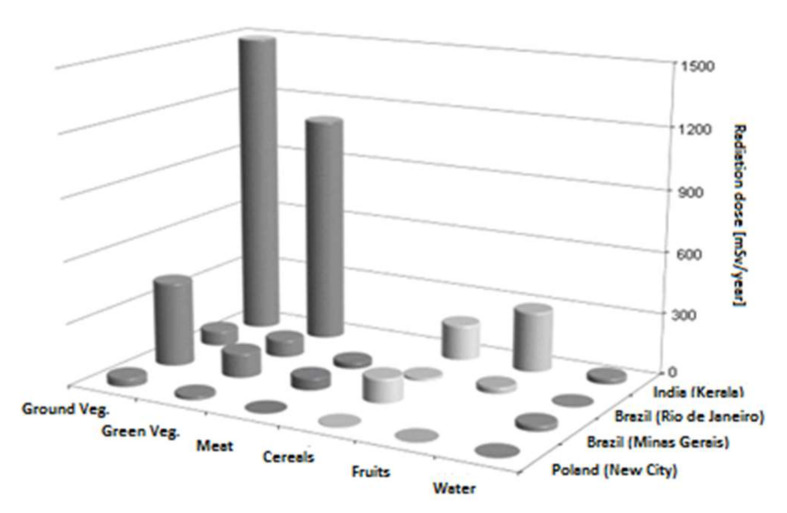
Annual radiation effective dose (µSv/y) for food consumers in India (Kerala), Brazil (Minas Gerais and Rio de Janeiro) and Poland.

The example of food showed that both the content of natural radionuclides in the soil and the eating habits of the local population have a direct impact on the amount of radioactive isotopes consumed through food. In some regions of India (Kerala), Iran (Ramsar) or Brazil (Minas Gerais), where the earth’s substance contains deposits rich in radium, uranium and thorium, food products contain increased ^238^U, ^232^Th, ^226^Ra, ^210^Pb and ^210^Po activity. This is confirmed by the large radiation doses received by consumers of ground and green vegetables in Kerala (above 0.9 Sv/y) and Minas Gerais (above 0.1 Sv/y) (Figure 2) [3,33,35,41].

For the average inhabitant of Poland, food and cigarette smoking are the main sources of absorption of ^210^Po and ^210^Pb in the human body (Table 1 and Table 2, Figure 1). In terms of food products, meat consumption is the source of over 40% (38 µSv/y) of ^210^Po but only 11% (5 µSv/y) of ^210^Pb. Fish consumption, especially, provides more than 30% (31 µSv/y) of the ^210^Po dose of the total food consumption and more than 80% of the dose for meat consumption. Poland is a country with a low consumption of fish (average 12 kg/y), but in some countries (Japan, Portugal, Norway and China) the consumption of marine fish, mussels and algae exceeds 60 kg per inhabitant per year (inhabitants absorb about 300 Bq ^210^Po), and they are the source of over 85% of the radiation dose that inhabitants of these countries receive [42]. Generally, the lower of the annual ^210^Po and ^210^Pb intakes are found in the countries where marine food plays a minor role in diets [1,43]. The activity concentration in different fish products (mackerel fillets, herring, sprat, tuna, sardines in oil, fish liver in oil) available on the Polish market fall into a very wide range from 0.07 to 27.1 Bq/kg for ^210^Po (average value is 5.16 Bq/kg) and from 0.07 to 0.88 for ^210^Pb (average value is 0.12 Bq/kg) (data unpublished). On the other hand, the mean concentration of both radionuclides in the fillet of Baltic fish (cod, herring and plaice) are: 0.35, 0.65 and 0.96 Bq/kg fresh weight for ^210^Po and 0.06, 0.15 and 0.23 Bq/kg fresh weight for ^210^Pb [44]. Taking into account the average consumption of fish in Poland at the level of 12 kg per year and the appropriate contribution (75% for fresh fish fillet and 25% for other fish products) of fresh fish fillets and canned fish products, we calculated the mean ingestion of ^210^Po and ^210^Pb to be 25.9 Bq and 1.7 Bq/y, respectively. The annual dose from this food source is 31.0 µSv for ^210^Po and only 1.2 µSv for ^210^Pb (Table 1). It follows that fish consumption is the most important source of both radionuclides in adult inhabitants of Poland and constitutes almost 30% of the total food dose derived from the decay of ^210^Po and ^210^Pb (Table 1).

In the case of mushrooms, individual consumption in Poland is on average 10 kg (fresh weight) per year. The average annual ingestion of the analyzed radionuclides was calculated on the basis of its concentration in mushrooms samples and equals 3.1 Bq for ^210^Po and 3.4 Bq for ^210^Pb (Table 1). The value of the effective dose of ^210^Po and ^210^Pb ingested by eating mushrooms is 14 µSv/y. In the case of the King Bolates species, which contains the most of both radionuclides, the dose value is much higher and exceeds 70 µSv. This value is slightly lower than that calculated for ^137^Cs as a result of the consumption of *Xerocomus badius* species [51].

Among other food products, the consumption of meat (43 µSv/y), fruit and vegetables (28.8 µSv/y), mushrooms (14 µSv/y), herbal teas (11.3 µSv/y only from decay of ^210^Po) and milk (7.2 µSv/y) have a significant contribution to the annual radiation dose of adults living in Poland. At the same time, the consumption of honey, alcohol and medicinal teas have a negligible contribution to the annual dose (below 1 µSv/y) (Table 1 and Table 2).

In summing up the research results, it should be stated that an adult Polish inhabitant receives an effective annual dose of 309 µSv from the inhalation and ingestion of ^210^Po and ^210^Pb. There are many potential other sources of polonium that the authors did not take into account in the publication, and differences in the way it enters the human body. Referring to the publications, [46] it can be noticed that the dose may differ for different species of fungi, the region of collection and the method of their preparation. In Poland, there is a tradition of picking fungi and the inhabitants pick their own fungi. They are not tested in any way prior to consumption. The authors of the above-mentioned publication suggest that radionuclide concentrations and doses may differ due to the above-mentioned factors. Our publication does not take into account regional differences. The situation is similar with regard to fish and their consumption. In Poland, there are fish products that come from many countries, contain various species of fish and are sold in various forms. Please note that in this case, we tried to summarize the doses of fish consumption for the inhabitants of Poland without paying attention to specific species [52]. Smoking is one of the main sources of radionuclides in human organism. The doses from smoking were determined on the basis of available data from Poland. In the case of tobacco data, doses may differ due to the calculation model used, which is sometimes corrected [26]. In the case of the use of cannabis and tobacco, they are the largest source of radiation dose from the studied isotopes. In terms of food, the consumption of fish, cereals, fruit and vegetables and fungi has the largest contribution in annual dose (Table 2). The amount of the calculated dose resulting from the decay of ^210^Po and ^210^Pb in the body is about 11% of the annual radiation dose received by a Polish inhabitant from natural sources (2.8 mSv) and is almost five times lower than the dose resulting from the inhalation of radon ^222^Rn [18] (Figure 1). Adults living in Poland receive similar or slightly higher doses of both radionuclides compared with the residents of other countries. For Japanese adult residents, the dose from their diet was estimated to be 50 µSv from ^210^Po and 53 µSv from ^210^Pb [53]. The lower values of the annual ^210^Po intakes are found in Argentina, Brazil and the UK (from 20 to 30 µSv/y), where the marine food plays a minor role in diets [43,49,54]. On the other hand, the highest annual ^210^Po intake (from 400 to 1200 µSv/y) is in countries like India, Portugal, Spain, the Marshall Islands, Lapland (in the north of Finland) and northern Canada, where marine food is an essential part of diet [4,55,56,57,58]. Unlike adult residents of other countries, adults living in Poland receive a high annual dose of ^210^Po and ^210^Pb as a result of smoking cigarettes and marijuana hash (162 µSv/y).

## 4. Conclusions

The research results presented in the paper showed that an adult Polish resident receives about an annual effective dose of 309 µSv of ^210^Po and ^210^Pb from inhalation and ingestion. The main source of both radionuclides in the body is smoking. This indicates that cigarettes and marijuana hash smoking and their absorption through the respiratory system are the main sources and the principal intake pathway of ^210^Po and ^210^Pb by Polish smokers. Cigarette and marijuana hash smoking could be the reason for the high incidence of cancer of all organs of the respiratory system in the Polish population. In terms of food, the sources of the dose from the tested isotopes are fish, grains, fruit and vegetables and mushrooms. The size of the calculated dose resulting from the decay of ^210^Po and ^210^Pb in the body is almost 1/10 of the annual radiation dose received by a Polish inhabitant from natural sources (2.8 mSv) and is almost five times lower than the dose resulting from the inhalation of ^222^Rn radon.

## Figures and Tables

**Table 1:** Annual ingestion and inhalation of ^210^Po and ^210^Pb by a Polish inhabitant as well as radiation dose.

Source	Annual Radiation Dose (µSv/y)	Percentage Contribution (%)
Water	0.9	0.3
Air	8.8	2.8
Cigarette and marijuana hash smoking	162	52.5
Marijuana tea	14	4.7
Food	123	40
Meat	43	14
Cereals, vegetables, fruit	45	14.6
Mushrooms	14	4.7
Teas (herbal, medical)	12	4
Milk	7	2.3
Others	1	0.3
Total	309	100

**Table 2:** Average annual radiation dose for an adult inhabitant of Poland from inhalation and ingestion of ^210^Po and ^210^Pb.

Intake Route	Annual Intake (Bq/y)		Radiation Dose (µSv/y)	Reference
	^210^Po	^210^Pb	^210^Po + ^210^Pb	
Air inhalation	1.63	3.08	5.4 + 3.4 = 8.8	[35]
Cigarette (smoking 1 pack per day)	35.2	38.3	116 + 42 = 158	[21]
Marijuana (*Cannabis sativa*)				[11]
hash	1.02	0.20	3.4 + 0.2 = 3.6	
tea	10.9	2.31	13.1 + 1.6 = 14.7	
Water	0.26	0.81	0.31 + 0.56 = 0.87	[35,36,37]
Food	73.4	51.2	88 + 35 = 123	
Milk	3.67	4.11	4.4 + 2.8 = 7.2	[45]
Cereals	8.00	9.35	9.6 + 6.5 = 16.1	[45]
Vegetables	10.3	9.37	12.4 + 6.6 = 19.0	[45]
Fruit	2.07	10.6	2.5 + 7.3 = 9.8	[45]
Meat	31.60	7.63	38 + 5 = 43	[45]
- Pork	4.16	4.41	5.0 + 3.0 = 8.0	[45]
- Beef	1.59	1.54	1.9 + 1.1 = 3.0	[45]
- Fish	25.85	1.68	31.0 + 1.2 = 32.2	unpublished data
Mushrooms	6.30	9.15	7.6 + 6.4 = 14.0	[46]
Honey	0.04	0.03	0.05 + 0.02 = 0.07	[47,48]
Herbal teas	9.45	-	11.3	[49]
Medical herbs	1.47	-	1.0	[50]
Alcohol				
- Wine	0.22	0.45	0.26 + 0.31 = 0.57	unpublished data
- Beer	0.31	0.52	0.37 + 0.36 = 0.73	[42]
Total	122.4	95.9	226 + 83 = 309	

1:. Annual radiation effective dose (µSv/y).

## Data Availability

Publicly available datasets were analyzed in this study. For each publication in the bibliography, the source of the data or the DOI is given.
